# The Association between Occupational Stress and Mental Health among Chinese Soccer Referees in the Early Stage of Reopening Soccer Matches during the COVID-19 Pandemic Outbreak: A Moderated Mediation Model

**DOI:** 10.3390/ijerph192416750

**Published:** 2022-12-13

**Authors:** Zongyu Liu, Liangyu Zhao, Shuzhen Wang, Yubo Gao, Liguo Zhang

**Affiliations:** School of Physical Education, Shandong University, Jinan 250061, China

**Keywords:** occupational stress, mental health, job burnout, perceived social support, soccer referees, COVID-19

## Abstract

Background: The sudden and unpredictable changes caused by the COVID-19 pandemic are a serious threat to the occupational stress and mental health of referees worldwide, which has not attracted widespread attention. The mental health of football referees has a certain influence on their job satisfaction or the accuracy of judgments. Methods: This study constructed a moderated mediation model to explore the buffer factors between occupational stress and mental health in Chinese soccer referees in the early stage of reopening soccer matches during the COVID-19 pandemic outbreak. Data from 317 Chinese soccer referees (aged 19–45) were collected through an online questionnaire in September–October 2022. Occupational stress, mental health, job burnout and perceived social support were measured, and moderated mediation model was analyzed. Results: The results of this study showed that occupational stress was negatively correlated with mental health through the mediating effect of job burnout and the moderated effect of perceived social support after controlling for demographic variables. Specifically, the association between occupational stress and mental health was weaker when perceived social support was higher and stronger. Conclusions: The results demonstrate that job burnout and perceived social support played important roles in buffering the negative effects of occupational stress on the mental health of Chinese soccer referees in the early stage of reopening soccer matches during the COVID-19 pandemic outbreak. The findings provide implications for mental health interventions in soccer referees during the public health crises.

## 1. Introduction

The novel coronavirus (COVID-19), which broke out in late 2019, has seriously threatened the physical and mental health of the global population [1]. It is an extremely difficult task for soccer referees to make the right judgement in a football match, who are often criticized for making bad decisions around the world, since it requires high physical and mental quality [2]. Comprehensive evidence has shown that the mental health of referees is closely related to their punishment behavior and will affect the effectiveness of their work [3]. In addition, the negative psychological impact due to the COVID-19 pandemic may increase the pressure on referees to make judgement. Radical and unpredictable changes during the pandemic may have led to a significant rise in occupational stress [4], which in turn has had a long-term negative impact on the well-being of referees and affected refereeing performance, as well as the development of competitive sports.

In the early days of the COVID-19 outbreak, most provinces activated public health emergency response measures and suspended some sports competitions [5]. As the prevention and control measures have gradually achieved good results, China has gradually begun to resume the operation of football matches and other events. However, the negative psychological impact of the pandemic on football referees still exists. When faced with challenges, many referees have a negative psychological state, which may be because job burnout [6] is involved in the process of occupational stress affecting mental health. According to the job demand–resource model, stressors consume physical and mental resources and could lead to health problems [7]. However, limited attention has been paid to the referee community in the context of the COVID-19 pandemic. Therefore, this study conducted a cross-sectional survey of Chinese soccer referees and constructed a moderated mediation model to investigate the mediating role of job burnout in the association between occupational stress and mental health of soccer referees. In addition, we examined the moderating effect of perceived social support on the relationship between occupational stress and mental health.

### 1.1. Theoretical Basis

The theoretical basis of the present study is the job demands–resources model (JD-R) [8]. The JD-R model was proposed by Demerouti et al., which revealed that work pressure would consume individual resources, thus leading to job burnout and health damage. This model expands the Effort–Reward Imbalance Model (REL) [9] and is a mainstream conceptual framework studying work stress, job burnout and mental health outcomes. It also has adaptability and applicability to many occupational environments. The JD-R model divides job characteristics into two categories: job demands and job resources [10]. Job demands involve occupational stress, which is associated with certain physical and psychological outcomes. Job resources include psychosocial resources such as perceived social support, and a high level of resources may bring higher motivation to work. According to this model, researchers can better understand and predict individual job burnout and mental health status. Therefore, this study conducted a theoretical analysis based on the JD-R model and proposed research hypotheses to investigate the relationship between occupational stress, job burnout, perceived social support and mental health among Chinese soccer referees.

### 1.2. The Role of Occupational Stress on Mental Health

Occupational stress adopted in this study refers to the stress caused by a particular individual in a particular job position [11], which can be classified into three classes, i.e., psychological stress, physical stress and behavioral stress. Soccer referees, as one of the focuses of attention in football matches, often experienced a rise in work pressure or deterioration of mental health due to the negative impact of the pandemic, which is not conducive to the smooth progress of football matches and the development of football careers. At present, few studies have focused on the impact of occupational stress on the mental health of football referees in the context of the COVID-19 epidemic, but it is certain that occupational stress is negatively correlated with mental health [12]. Research has shown that soccer referees at amateur and elite levels experience various forms of stressors, which have a negative impact on their mental health [13]. Moreover, Webb’s research suggests referees will be required to spend extended periods of time away from home while soccer matches resume, and the uncertainty over their earnings may cause them a certain amount of occupational stress, negatively affecting their mental health and well-being [14]. A large number of studies have shown that excessive time pressure has a negative impact on the mental health of groups in different occupations [15,16,17], which are particularly vulnerable to adverse environments. For example, Roxburgh [18] found that individual depression level will increase with the increase of time pressure and produce negative emotions. Scholars have studied the relationship between occupational stress and mental health in different groups. Schilling et al. found that occupational stress was negatively correlated with the mental health of police officers, and concluded that occupational stress may be more closely related to mental health than physical health [19]. Perceived occupational stress has been reported to have adverse and pessimistic effects on the mental health of primary school teachers, such as insomnia, anxiety, happiness, etc. [20]. Therefore, based on the perspective of positive psychology, this study examines how occupational stress affects referees’ mental health and identifies mediating and moderating factors that may mitigate the harmful consequences of occupational stress. Based on the results of previous studies, we propose a hypothesis that occupational stress will negatively predict the mental health of Chinese football referees during the COVID-19 pandemic (H1).

### 1.3. The Mediating Role of Job Burnout between Occupational Stress and Mental Health

Job burnout refers to the state of physical and mental fatigue and exhaustion of individuals under the pressure of work, which is the result of an individual’s failure to cope with pressure [21]. According to the demand–resource model of work [8], stressors will consume an individual’s physical and mental resources, which will lead to mental health problems. Thus, burnout has been found to reinforce the harmful consequences of adverse circumstances. The outbreak of the COVID-19 pandemic has undoubtedly brought a certain degree of pressure to referees. The important role of job burnout is supported by a large amount of evidence on the relationship between stress and mental health. Moreno Fortes et al. found that occupational stress is a risk factor for mental health, and job burnout plays an intermediary role in the process of occupational stress affecting mental health [22]. Numerous studies have documented the detrimental effects of job burnout on an individual’s mental health. For example, Lu et al. confirmed that job burnout is a risk factor for individual mental health, and taking measures to alleviate individual job burnout is an important way to improve mental health [6]. Similarly, scholars have found that job burnout can have very serious consequences for an individual’s physical and mental health, including insomnia and various mental health problems [23,24]. The positive correlation between stress and burnout has also been confirmed. Research shows that individuals under long-term high pressure will experience job burnout, resulting in low work efficiency, emotional loss and other problems, which will affect their behavior [25]. Evidence on prior factors of burnout suggests that occupational stress plays a key role as a predictor of burnout, especially for adults [26,27]. Occupational stress has been found to promote the development of job burnout and cause a range of psychological problems [6]. Therefore, based on empirical and theoretical evidence, we propose the hypothesis that occupational stress and mental health are associated with job burnout. In other words, job burnout played a mediating role between referees’ occupational stress and mental health during the pandemic (H2).

### 1.4. The Moderating Effect of Perceived Social Support on the Relationship between Occupational Stress and Mental Health

Although the mediating effect can reveal the internal mechanism of how the independent variable affects the dependent variable, it fails to explain how the association changes under different conditions. The association between occupational stress and mental health has been supported by a large amount of evidence; however, knowledge about the moderating variables between them is still limited. Therefore, it is important to explore the moderating variables between occupational stress and mental health. In addition, in the current context, it helps to understand the psychological characteristics of referees in the early stage of reopening soccer matches during the COVID-19 pandemic. The perspective of positive psychology emphasizes the role of positive intrinsic factors in challenging situations, where the perception of the protective effect of social support is widely supported, especially in studies of adults [28]. Perceived social support is defined as the influence that a person obtains through social connections [29] that can reduce psychological stress, relieve tension, and improve social adaptability. Perceived social support has been shown to be an important predictor and source of mental health [30]. According to a previous study [31], when people face stressful events, they will mobilize resources to improve the situation and offset the ongoing challenges, thus reducing the negative effects of stress. The loss or gain of resources is an important mechanism driving the stress response. Previous research has found that individuals with higher levels of social support are likely to experience lower negative psychological emotion even if job factors (such as stress) negatively affect their mental health [32]. Research by Kilic et al. investigated the association between psychosocial stressors and mental health symptoms among soccer referees, and found that poor satisfaction with social support in this group was associated with adverse mental health symptoms [33]. Wang et al. found that social support can moderate the effect of stress on adverse psychological conditions. In addition, social support is a psychosocial resource that has also been shown to moderate the relationship between stress and depression [34]. Therefore, our model focuses on understanding the moderating role of perceived social support in the association between occupational stress and mental health. Based on the positive nature of perceived social support, we hypothesize that there is a positive correlation between perceived social support and mental health, and that referees with higher levels of perceived social support had a lower correlation between occupational stress and mental health (H3).

### 1.5. The Present Study

Research on the mental health status of soccer referees is limited and mainly concentrated in Europe and Egypt. In addition, the relatively late resumption of soccer matches in China could have a worse effect on referees’ mental health. Therefore, it may be valuable to conduct research on the mental health of soccer referees in China. Based on empirical and theoretical evidence, this study constructed a moderated mediation model to examine the mediating effect of job burnout on the relationship between occupational stress and mental health of referees, and to examine the moderating effect of perceived social support in the direct effect path. From a positive psychology perspective, this model could further influence the reduction of the negative impact of environment-related stress on referees’ mental health, and help to develop effective interventions to promote referees’ mental health during the gradual opening of matches in a pandemic. The following are the hypotheses of this study:

**Hypothesis (H1):**  *Occupational stress is negatively correlated with referees’ mental health.*

**Hypothesis (H2):**  *Job burnout plays a mediating role in the association between occupational stress and mental health.*

**Hypothesis (H3):**  *The association between occupational stress and mental health was moderated by perceived social support and the relationship was weaker among referees with higher levels of perceptive social support.*

**Figure 1:**
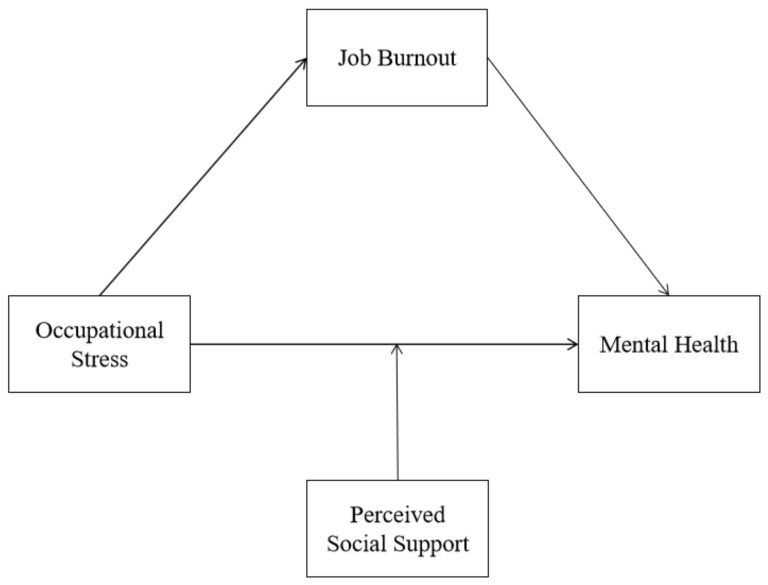
Hypothetical model of the association among occupational stress, mental health, job burnout, and perceived social support.

The conceptual model is shown in Figure 1.

## 2. Methods

### 2.1. Data Collection

The study protocol, which included a series of questionnaires for Chinese soccer referees in the early stage of reopening soccer matches during the COVID-19 pandemic outbreak, was approved by the Ethics Committee of Shandong University (No. ECSBMSSDU2022-1-086). Cross-sectional surveys were conducted across China from September to October 2022. During the study period, the COVID-19 epidemic in China was brought under certain control, but new confirmed cases of COVID-19 and the negative impact of the epidemic still existed. Due to pandemic control measures, face-to-face interviews with all referees scattered across the country were not possible during the survey. This study was conducted through an online questionnaire platform (www.wjx.com, accessed on 1 August 2022), which is a commonly used online survey channel in China during the pandemic. For referees who could conduct face-to-face surveys, the researchers conducted a questionnaire survey by showing the link of the questionnaire on-site in a face-to-face manner, and explained the questions that were not thoroughly understood by the participants. For the online survey, the researchers used telephone communication to address questions that participants faced.

The referees volunteered to take part by clicking on a link to a questionnaire presented or sent by the researchers. A total of 344 Chinese soccer referees completed the survey from September to October 2022. Data screening met the following criteria: (1) participants voluntarily participated in the survey after reading the instructions; (2) the entire investigation was completed as required; (3) the respondents qualified as Chinese football referee Level 3 or above; (4) the time to complete the questionnaire was consistent with the estimate (10 min; surveys completed within a shorter period of time were deleted). The final sample included 317 respondents, 265 of whom were male (54.1%) and 52 of whom were female (45.9%), indicating a high proportion of male soccer referees in China. Participants ranged in age from 19 to 45. Although there has been no large-scale study based on a representative sample of global soccer referees, the sample in this study is from a wide range of coverage in China. Our sample seems comparable to previous studies on referees, healthcare workers or other groups with relatively high occupational stress levels. Therefore, the sample is highly representative of the target population (referees).

### 2.2. Variables and Measures

#### 2.2.1. Occupational Stress

The Chinese version of the Effort–Reward Imbalance Scale (ERI) was used to investigate the occupational stress of Chinese football referees [35]. The ERI was developed by Siegrist [36] and is a widely used and relatively standardized questionnaire in China, and has proved sufficient reliability and validity in the sample population in China [37,38]. The ERI questionnaire contains 3 dimensions of effort, reward and overcommitment, with 6, 11 and 6 items, respectively, and a total of 23 items. The values assigned to the dimension items of effort and reward are L-5 points, among which 1 point represents disagreeing with the setting of the corresponding item. The values of 2 to 5 points represent the degree of trouble with the setting of the corresponding item, which are not at all, somewhat, troubled and very troubled, respectively. The values assigned to the intrinsic contribution dimension are 1–4 points, representing completely disagree, disagree, agree, and completely agree with the setting. The sum of the scores of each item in each dimension is the total score of the dimension. The study measured occupational stress levels as the effort: reward ratio, which is calculated by dividing effort by reward and then multiplying by 11/6 to correct for differences in the number of items in both dimensions. In this study, the Cronbach’s alphas for the extrinsic effort, reward and overcommitment subscales were 0.854, 0.856, and 0.758, respectively.

#### 2.2.2. Mental Health

The Chinese version of the Depression Anxiety Stress Scale-21 [39] (DASS-21), which was originally [40] developed by Lovibond et al., was used to measure the mental health status of Chinese football referees, with a total of 21 items, including three subscales of depression, anxiety and pressure. Higher subscale scores indicate higher levels of negative symptoms such as depression, anxiety and stress. The pressure dimension of this scale refers to a negative psychological and emotional condition. Each subscale contains 7 items, and the Likert 4-level scoring method was adopted, with 0 to 3 points representing “not consistent”, “sometimes consistent”, “often consistent” and “always consistent”, respectively. The higher the score is, the more serious the symptoms are. When the scores on the subscale are added together, higher scores indicate lower levels of mental health. The scale has been proven to have good reliability and validity in Chinese population [41]. In this study, the Cronbach’s alphas for depression, anxiety and pressure were 0.910, 0.890, and 0.904, respectively.

#### 2.2.3. Job Burnout

The Chinese version [42] of the Maslach Burnout Inventory–General Survey (MBI-GS) was used to measure the job burnout status of football referees. The questionnaire was originally developed by Maslach [43] and has 22 items in 3 dimensions, which are low sense of accomplishment (8 items), emotional exhaustion (8 items) and depersonalization (6 items), respectively. The Likert 7-level scoring method was adopted, and 0 ~ 6 points were used to represent the score range of individual feelings; 0 points are “never” and 6 points are “everyday”. The two dimensions of emotional exhaustion and depersonalization were scored in a positive way, while the dimension of low sense of accomplishment was scored in a reverse way. The score of each dimension was the sum of scores of all items in the dimension divided by the number of items. The higher the score, the higher the level of job burnout. The scale has been verified to have good reliability and validity in Chinese population [44]. In this study, the Cronbach’s alphas for low sense of accomplishment, emotional exhaustion and depersonalization were 0.961, 0.855, and 0.812, respectively.

#### 2.2.4. Perceived Social Support

The Chinese version of the Perceptive Social Support Scale (PSSS) was selected to measure the level of perceived social support [45]. The original version [46] was compiled by Zimet et al. The scale consists of 12 items, including three dimensions of family support, friend support and other support. A 7-level score was used, and the higher the score, the higher the degree of social support felt by the individual. This scale has been widely used in the study of Chinese population and has been proven to have good reliability and validity [47,48]. The Cronbach’s α coefficient of the scale in this study was 0.962.

### 2.3. Statistical Analysis

IBM SPSS version 22.0 (IBM, Armonk, NY, USA) and plug-in PROCESS Macro version 3.3 for SPSS (IBM Corporation, Armonk, NY, USA) [49] were used for data analysis. The bootstrap method was used to test the significance of regression coefficients. Descriptive statistics and correlations among variables were assessed. Both the mediation effect of job burnout and the moderating role of perceived social support were tested, respectively, using Model 4 and Model 59 of the PROCESS macro for SPSS. Model 4 was used to test the mediating role (H2) of job burnout along with the direct relationship between occupational stress and mental health. Model 59 was used to simultaneously test whether the perceived social support moderated the relationship between the direct effect of occupational stress on mental health (moderator hypothesis, H3). An effect was deemed significant when the 95% confidence interval (CI) did not include zero, based on a bootstrap random sample (*n* = 5000). Moreover, we used the structural equation model (SEM) made by AMOS 22.0 software to verify the proposed model. Descriptive fit indices were utilized to determine model fit in our research. The descriptive fit indices consist of the CFI, the TLI, RMSEA and the SRMR, which have most commonly been studied as indicators of structural equation modeling and confirmatory factor analyses [50]. In the current research, we followed the approach of Accurso et al. (2013), which represents a good fit (or well-fitting) model by CFIs and TLIs ≥ 0.95 (0.90 -- 0.94), RMSEA < 0.05 (to 0.08), and SRMR < 0.05 (to 0.08) [51]. If three of the four descriptive indicators indicate a good fit, the model is determined to be well-fitting. All variables were standardized before entering the mediation model.

## 3. Results

### 3.1. Test for Common Method Bias

Univariate tests were used to test for common methodological bias due to self-reporting in this study. The results showed that the common factor explained 26.265% of the total variance, which was lower than the threshold of 40%. Therefore, to a certain extent, there is no common method bias in this study.

### 3.2. Descriptive Statistics and Correlational Analysis

All variables are described and correlated in Table 1. The basic descriptive data for occupational stress, mental health, job burnout, and perceived social support showed that the mean total scores for occupational stress were 0.22 ± 0.11 (range = 0.06–1.49), the mean total scores for job burnout were 42.52 ± 12.26 (range = 22–110), and the mean total scores for perceived social support were 65.12 ± 13.77 (range = 12–84). Moreover, the mean total scores for pressure, anxiety and depression were 2.65 ± 3.36 (range = 0–21), 2.06 ± 2.94 (range = 0–21), and 1.96 ± 2.98 (range = 0–21), respectively. However, in our sample, referees with mild or above stress symptoms, mild or above anxiety symptoms, and mild or above depression symptoms accounted for 5.9%, 22.7% and 16.1%, respectively. The correlational analysis results showed that occupational stress was negatively correlated with perceived social support, but positively correlated with job burnout, depression, anxiety and pressure. Meanwhile, job burnout was positively correlated with depression, anxiety and pressure. In addition, age was significantly correlated with job burnout and anxiety, and BMI was significantly correlated with anxiety.

### 3.3. Tests of Moderated Mediation

**Figure 2:**
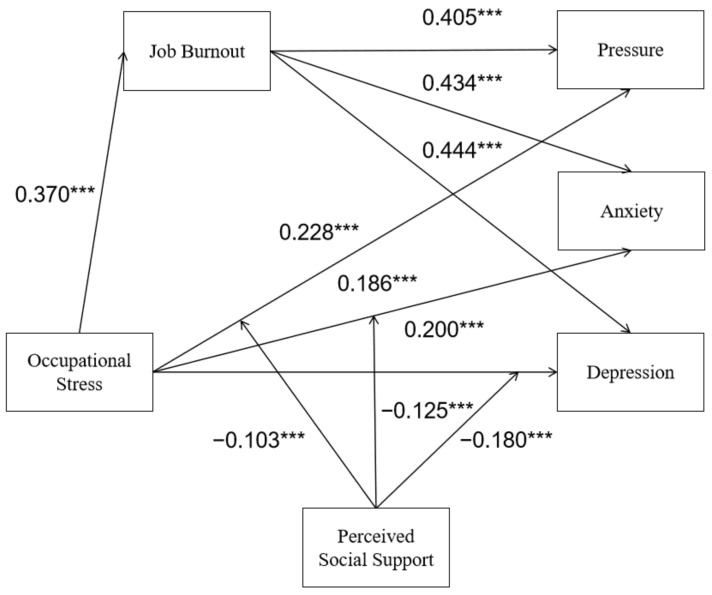
Moderated mediation model of job burnout and perceived social support in the association between occupational stress and mental health. *** *p* < 0.001.

After the data were standardized, Model 59 in the PROCESS was used to test the moderated mediation model, in which occupational stress was the independent variable, job burnout was the mediating variable, and the three dimensions of mental health were the dependent variable. In addition, perceived social support was incorporated into a three-dimensional pathway from occupational stress to mental health. The structural equation model was used to verify the model, and the fitting index was: χ²/df = 4.7. CFI = 0.97; TLI = 0.92; SRMR = 0.06; RMSEA = 0.11, indicating that the model had an acceptable fitting effect. The results are consistent with H1 and H2. Occupational stress had a negative predictive effect on perceived social support, and a positive predictive effect on job burnout, depression, anxiety and pressure. The interaction between occupational stress and perceived social support negatively predicted mental health. In addition, job burnout was positively correlated with the dimensions of depression, anxiety and stress (Table 2). These results suggest that occupational stress, perceived social support, job burnout and mental health constitute a moderated mediating model, with job burnout mediating between occupational stress and mental health, and perceived social support moderating in the direct path of the model (Figure 2). In the sampling process, the significance of the mediation effect was tested by the bootstrap method. All upper and lower limits of 95% confidence interval did not contain 0, indicating that both direct and indirect effects were significant. 

**Figure 3:**
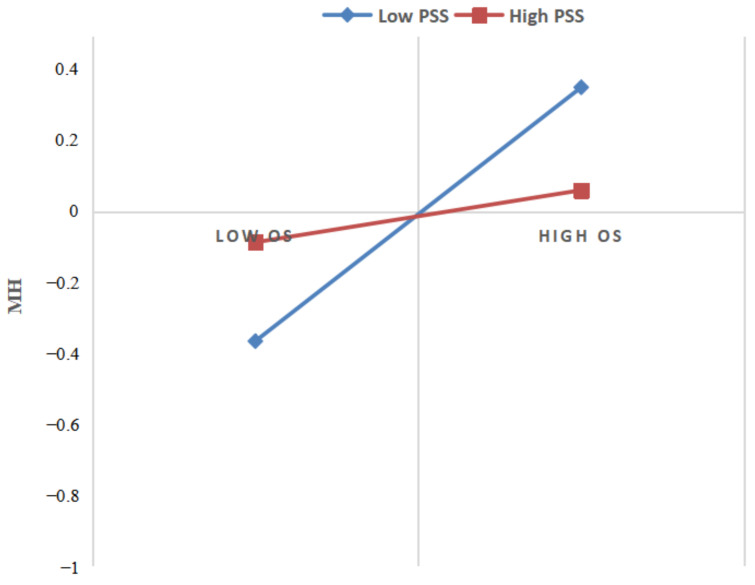
Simple slopes of occupational stress and mental health for referees with low and high levels of perceived social support. OS: occupational stress; PSS: perceived social support; MH: mental health.

The combination of the three dimensions of mental health reflects the overall mental health status of the individual. Figure 3 shows the relationship between occupational stress and mental health at two levels of perceptive social support (M + 1SD and M − 1SD). In order to facilitate and understand the interpretation of the moderating effect of perceptive social support intuitively, we presented the overall level of mental health in Figure 3. The simple slope test showed that the occupational stress of referees with low perceived social support (M − 1SD) and high perceived social support (M + 1SD) was negatively correlated with their mental health. Bias-corrected bootstrap analyses further showed that occupational stress and depression (β = 0.345, SE = 0.047, 95%CI = [0.254, 0.437]), anxiety (β = 0.270, SE = 0.051, 95%CI = [0.169, 0.371]), and pressure (β = 0.329, SE = 0.051, 95%CI = [0.229, 0.429]). For referees with high levels of social support awareness, occupational stress and depression (β = 0.045, SE = 0.067, 95%CI = [−0.086, 0.176]), anxiety (β = 0.098, SE = 0.073, 95%CI = [0.050, 0.280]), and pressure (β = 0.120, SE = 0.073, 95%CI = [−0.023, 0.263]) were weaker. Thus, perceived social support moderates the association between occupational stress and mental health, which supports hypothesis 3 (H3).

## 4. Discussion

The current study focused on the association between occupational stress and mental health among Chinese soccer referees during the resumption of soccer matches following the outbreak of the COVID-19 pandemic. A model with job burnout as the mediating variable of the relationship between occupational stress and mental health and perceived social support as the moderating variable was constructed. Analysis based on data from an online survey of 371 Chinese soccer referees supports the model. Our findings suggest that occupational stress during the resumption of football matches following the outbreak of the COVID-19 pandemic had a negative impact on the mental health of Chinese football referees. At the same time, job burnout mediated the relationship between occupational stress and mental health, while perceived social support moderated the relationship between occupational stress and mental health, both of which could serve as a buffer.

### 4.1. Effect of Ocupational Stress on Mental Health

The results of correlation analysis showed that occupational stress was positively correlated with poor mental health conditions (such as depression, anxiety and pressure). Therefore, the occupational stress associated with the pandemic can positively predict the adverse psychological status of Chinese football referees, that is, the positive prediction of mental health, which supports H1. As a special occupational group, referees may be more vulnerable to the negative psychological consequences of insecurity caused by the pandemic as COVID-19 has negatively impacted the mental health and well-being of adults [52]. During the outbreak of the COVID-19 pandemic, referees (both at the elite level and the grassroots level) have faced considerable challenges that remain after the easing of lockdown measures and the gradual resumption of soccer matches [33]. In reality, with the gradual resumption of football matches, referees are facing various pressures, which pose more severe challenges to their work. The current findings suggest that occupational stress among referees in the early stage of reopening soccer matches during the COVID-19 pandemic led to higher levels of depression, anxiety and pressure, indicating a negative impact on mental health. This negative effect is consistent with other studies conducted at the same time that show links between occupational stress, depression, anxiety, pressure and other mental health conditions [11,18,19,22,53,54,55]. In addition, the current findings contribute to understanding the impact of occupational stress during the initial resumption of football matches on mental health in the context of the COVID-19 pandemic, suggesting that occupational stress was a key factor in the deterioration of referees’ mental health during the pandemic.

### 4.2. The Mediating Role of Job Burnout

According to the Person–environment Fit Theory (P-E Fit Theory) [56], stress comes from congruence between the individual and the environment. The sudden outbreak of the pandemic has caused certain psychological distress to individuals. Meanwhile, some measures [57] taken by the country in response to the epidemic (such as strict control of the activity path of referees) may bring pressure to referees from both internal and external levels. This study confirms the mediating role of job burnout between adolescents’ perceived insecurity and subjective well-being. Specifically, the analysis shows that occupational stress is positively correlated with job burnout, while job burnout negatively predicts mental health, showing a significant mediating effect. Therefore, referees’ occupational stress can affect their mental health by enhancing their sense of job burnout. At the same time, the mediating of burnout can also serve as a buffer for its relationship, thus supporting H2. Our research is also consistent with the JD-R model. Referees’ emotional exhaustion is the primary reaction to job stressors [58], and the sense of job burnout may also have a certain relationship with the management style of superior managers. With the reform and development of Chinese football, higher requirements are put forward for referees, which is also one of the reasons for referees’ job burnout. Previous research has also confirmed that occupational stress is significantly associated with job burnout in different groups, with long-term negative effects on their mental health growth [6,22,23,59,60,61]. Studies have also confirmed the mediating effect of job burnout between occupational stress and mental health [21]. Our model further demonstrates that environment-related occupational stress extends the exploration of occupational stress and emotional exhaustion by increasing the negative impact of burnout on referees’ mental health during the pandemic. In addition, burnout can act as an important buffer factor in the relationship between occupational stress and referees’ mental health. Based on the negative characteristics of job burnout, reducing the job burnout of referees can help them deal with the epidemic more rationally and have a positive attitude toward the work of football matches. This helps mitigate the impact of occupational stress during the pandemic; as a result, it helps stabilize their mental health. Consistent with the findings of previous studies, this study suggests that occupational stress may reduce the mental health status of individuals by increasing the level of burnout. Our results further support the protective effect of reduced burnout on referees’ mental health in the context of the COVID-19 pandemic. Therefore, improving burnout through psychological intervention during the pandemic may be an important way to help improve the mental health of referees.

### 4.3. The Moderating Role of Perceived Social Support

In addition to the cushioning effect of job burnout, the important role of social support as internal resource satisfaction cannot be ignored, especially in the unpredictable and adverse circumstances of a pandemic that has only recently allowed football matches to be re-opened. Less research has been conducted to understand the moderating effects of social support [61] on the relationship between environment-related occupational stress and individual mental health, especially in the referee community. Therefore, it is of great significance to explore the moderating effect of social support in this study, which fills the gap of previous studies. Our study found that perceptive social support had a moderating effect on the relationship between occupational stress and referees’ mental health, and the moderating effect occurred on the direct path of the mediating pathway, which supported hypothesis H3 and was consistent with the JD-R model. The relationship between occupational stress and adverse psychological conditions weakened when referees’ perceived social support levels increased. When referees have a high level of awareness of social support, the specific context of COVID-19 causes fewer negative mental health conditions, because support resources can mitigate the effects of stress by providing problem-solving strategies such as material support and intangible resources [62]. These results also support the conclusions of the social support buffer model in previous studies [63]. This suggests that improving the social support level of referees can help improve their mental health. In addition, according to self-determination theory [64], the satisfaction of relational needs is the basic nutrient for individual growth. Individuals with high levels of occupational stress who perceive others as providing important resources can help them redefine the potential harm caused by stress [34,65]. Resources from society can help individuals to meet their basic psychological needs, so that they can cope with the adverse effects of stress with a fuller spirit, thus improving their mental health [66]. The results of this study are also consistent with previous studies [31,67]. According to the research results, perceived social support is a protective factor for referees’ mental health improvement, suggesting that all sectors of society should invest more resources and energy to provide various kinds of support for referees, so as to relieve their occupational pressure and improve their mental health condition.

### 4.4. Implications

The findings of this study have important theoretical implications. First, the study aims to investigate football referees in the early stage of reopening soccer matches during the specific phase of the COVID-19 pandemic, and to extend the study to occupational stress in the wider population. Previous studies mainly focused on the occupational stress of medical staff, teachers, police and other groups, but there are few studies to understand the impact of occupational stress on referees’ mental health [13,14,15]. Second, our study enriches and expands the research on mental health. Thirdly, we constructed a moderated mediation model, focusing on the mediating role of job burnout and the moderating role of social support. This model can provide important empirical evidence for understanding the mechanism of occupational stress reduction on referees’ mental health in the early stage of reopening soccer matches during the COVID-19 pandemic. In addition, the results have important practical implications. Mental health has received a lot of attention as the COVID-19 pandemic has progressed. This study, from the perspective of positive psychology, is of great significance for formulating intervention strategies to improve referees’ mental health, especially new ideas to promote mental health by reducing job burnout and increasing perceptive social support. The results of this study are expected to have a significant impact on future practice when determining how to protect referees from the negative effects of the pandemic on their mental health. Referees can prevent the negative psychological effects of depression, stress, anxiety and other reactions to the novel coronavirus by feeling and understanding the practical social support they receive. The more social support they feel and the less burnout they experience, the more they can adjust and improve their physical and mental health. Alleviating negative emotions in response to public health emergencies has a positive impact on their physical and mental health.

### 4.5. Limitations and Future Research Directions

In addition to the important theoretical and practical significance, this study also has some limitations, which need to be further explored in the future. Firstly, this study mainly explored the moderated mediation model based on the data of Chinese soccer referees collected during the first football competition period after the outbreak of the COVID-19 pandemic. Therefore, the generalizability of these findings in other areas remains to be explored. According to the WHO data, during the period of our investigation, China overcame the critical phase of the worst of the COVID-19 pandemic, with the national outbreak under initial control. However, cases are still increasing in other countries around the world. Previous studies have shown that cultural background or the severity of the epidemic can have an important impact on an individual’s mental health or feelings of burnout. Therefore, considering the development trend of worldwide COVID-19 in different countries of different cultural background and related measures, some countries may not be able to resume football matches, people of different areas are likely to experience different levels of occupational stress or mental stress reactions, and psychological health is not affected to the same degree. Therefore, considering the above factors, the applicability of this model in other fields in Asia or globally still needs to be further verified. Secondly, this study mainly explored the mediating effect of job burnout and the moderating effect of perceived social support between occupational stress and mental health of Chinese football referees, which was supported by existing evidence and data analysis. However, a review of previous studies suggests that other variables may mediate or moderate the relationship between occupational stress and mental health. Therefore, it is of interest to further investigate the role of hope in future studies of different groups and cultural contexts. Considering that the average occupational stress level of referees in this study is not high, it is likely that there are other pathways through which increased job burnout influences mental health. Finally, it is difficult to identify causal effects with cross-sectional designs, and longitudinal investigations may help to improve the understanding of changes in referees’ mental health throughout the outbreak of different pandemics and enhance the reference value of theoretical applications.

## 5. Conclusions

The study was conducted starting in September 2022, during the reopening of football matches after a suspension due to COVID-19 lockdowns, using data from an online questionnaire conducted with 317 Chinese football referees. As for the study’s main findings, first, occupational stress during the post-pandemic resumption of football had a negative predictive effect on referees’ mental health. The negative relationship between job stress and mental health was mediated by job burnout, and perceived social support can moderate the direct path of the mediation model. The higher the level of social support perceived by football referees, the weaker the link between occupational stress and mental health, and the higher their mental health. The findings suggest that burnout and perceived social support play an important role in cushioning the negative effects of occupational stress on the mental health of soccer referees.

## Figures and Tables

**Table 1:** Means, standard deviation and correlations of variables.

Variables	M	SD	1	2	3	4	5	6	7	8	9	10
1 Gender ^a^	-	-	1									
2 Age ^b^	-	-	−0.247 **	1								
3 Level of education ^c^	-	-	0.034	0.120 *	1							
4 BMI	22.38	2.85	−0.432 **	0.455 **	−0.033	1						
5 Occupational Stress	0.22	0.11	−0.106	0.083	0.068	0.018	1					
6 Job Burnout	42.52	12.26	0.056	−0.142 *	0.043	−0.122 *	0.413 **	1				
7 Perceived Social Support	65.12	13.77	−0.010	0.049	−0.015	0.007	−0.169 **	−0.393 **	1			
8 Pressure	2.65	3.36	0.089	−0.086	0.005	−0.089	0.458 **	0.503 **	−0.164 **	1		
9 Anxiety	2.06	2.94	0.064	−0.121 *	0.057	−0.096	0.425 **	0.524 **	−0.190 **	0.869 **	1	
10 Depression	1.96	2.98	0.072	−0.082	−0.028	−0.088	0.495 **	0.568 **	−0.238 **	0.861 **	0.832 **	1

Note: ^a^ (0 = female, 1 = male), ^b^ (1 = 19–25 years old, 2 = 26–35 years old, 3 = 36–45 years old) and ^c^ (1 = High school diploma or below, 2 = junior college or college degree, 3 = Master’s Degree, 4 = Doctoral Degree) are dummy variables. ** *p* < 0.01, * *p* < 0.05.

**Table 2:** Testing for moderated mediation effects of job burnout and perceived social support.

Regression Equation		Goodness of Fit			Significance			
Outcome	Predictor	*R*	*R^2^*	*F*	*β*	*se*	*t*	95%CI
Job Burnout	Occupational Stress	0.528	0.279	40.335 ***	0.370	0.054	6.907	(0.264, 0.475)
	Perceived Social Support				−0.331	0.049	−6.790	(−0.427, −0.235)
	Occupational Stress*Perceived Social Support				0.020	0.035	0.564	(−0.049, 0.088)
Pressure	Occupational Stress	0.596	0.355	34.222 ***	0.228	0.055	4.171	(0.120, 0.335)
	Job Burnout				0.405	0.054	7.535	(0.299, 0.510)
	Perceived Social Support				0.014	0.053	0.266	(−0.090, 0.118)
	Occupational Stress*Perceived Social Support				−0.125	0.036	−3.446	(−0.196, −0.054)
	Job Burnout*Perceived Social Support				0.064	0.049	1.319	(−0.032, 0.160)
Anxiety	Occupational Stress	0.588	0.345	32.805 ***	0.186	0.055	3.388	(0.078, 0.295)
	Job Burnout				0.434	0.054	8.020	(0.328, 0.540)
	Perceived Social Support				0.010	0.053	0.180	(−0.095, 0.114)
	Occupational Stress*Perceived Social Support				−0.103	0.037	−2.818	(−0.175, −0.031)
	Job Burnout*Perceived Social Support				0.018	0.049	0.370	(−0.078, 0.115)
Depression	Occupational Stress	0.679	0.461	53.135 ***	0.200	0.050	4.003	(0.102, 0.298)
	Job Burnout				0.444	0.049	9.034	(0.347, 0.540)
	Perceived Social Support				−0.034	0.048	−0.695	(−0.129, 0.061)
	Occupational Stress*Perceived Social Support				−0.180	0.033	−5.421	(−0.245, −0.115)
	Job Burnout*Perceived Social Support				0.031	0.045	0.694	(−0.057, 0.119)

Note: ***p < 0.001.

## Data Availability

Data are available on request from the corresponding author.
